# Long-Term Neonatal EEG Modeling with DSP and ML for Grading Hypoxic–Ischemic Encephalopathy Injury

**DOI:** 10.3390/s25103007

**Published:** 2025-05-10

**Authors:** Leah Twomey, Sergi Gomez, Emanuel Popovici, Andriy Temko

**Affiliations:** Electrical and Electronic Engineering Department, University College Cork, T12 K8AF Cork, Ireland; sgomez@umail.ucc.ie (S.G.); e.popovici@ucc.ie (E.P.); atemko@ucc.ie (A.T.)

**Keywords:** HIE, background grading, machine learning, signal processing, sonification, neonates

## Abstract

Hypoxic–Ischemic Encephalopathy (HIE) occurs in patients who experience a decreased flow of blood and oxygen to the brain, with the optimal window for effective treatment being within the first six hours of life. This puts a significant demand on medical professionals to accurately and effectively grade the severity of the HIE present, which is a time-consuming and challenging task. This paper proposes a novel workflow for background EEG grading, implementing a blend of Digital Signal Processing (DSP) and Machine-Learning (ML) techniques. First, the EEG signal is transformed into an amplitude and frequency modulated audio spectrogram, which enhances its relevant signal properties. The difference between EEG Grades 1 and 2 is enhanced. A convolutional neural network is then designed as a regressor to map the input image into an EEG grade, by utilizing an optimized rounding module to leverage the monotonic relationship among the grades. Using a nested cross-validation approach, an accuracy of 89.97% was achieved, in particular improving the AUC of the most challenging grades, Grade 1 and Grade 2, to 0.98 and 0.96. The results of this study show that the proposed representation and workflow increase the potential for background grading of EEG signals, increasing the accuracy of grading background patterns that are most relevant for therapeutic intervention, across large windows of time.

## 1. Introduction

Hypoxic–Ischemic Encephalopathy (HIE) describes the brain injury suffered by neonates due to a critical shortage of blood or oxygen supply to the brain directly before, during, or after birth [1]. Different levels of neurological impairment occur dependent on the severity level of the injury [2]. Long-term neurological impairments affect 25–30% of neonates with HIE [3], including cerebral palsy, epilepsy, hearing loss, visual defects, and cognitive impairments [4,5]. An incidence rate of 1–8 babies per 1000 births and 26 babies per 1000 live births have been reported for HIE in developed and developing countries, respectively [1,6,7].

Significant research has gone into optimal treatment plans for patients with HIE, each with the goal to minimize long-term effects, with therapeutic hypothermia being the most prominent treatment at present [8]. This treatment has been found to be very effective, with mortality rates decreasing from 27% to 12.8% after implementing this treatment protocol as in [9]. While the treatment is effective, optimal results for therapeutic hypothermia to treat HIE have been noted when initiated within Phase I post-injury [10]. Phase I describes the initial onset of energy failure, which occurs due to the shortage of oxygen or blood supply to the neonate [10], and is usually defined as the first 6 h post-injury. The effectiveness of the treatment significantly decreases when administered outside this window [11].

**Figure 1:**
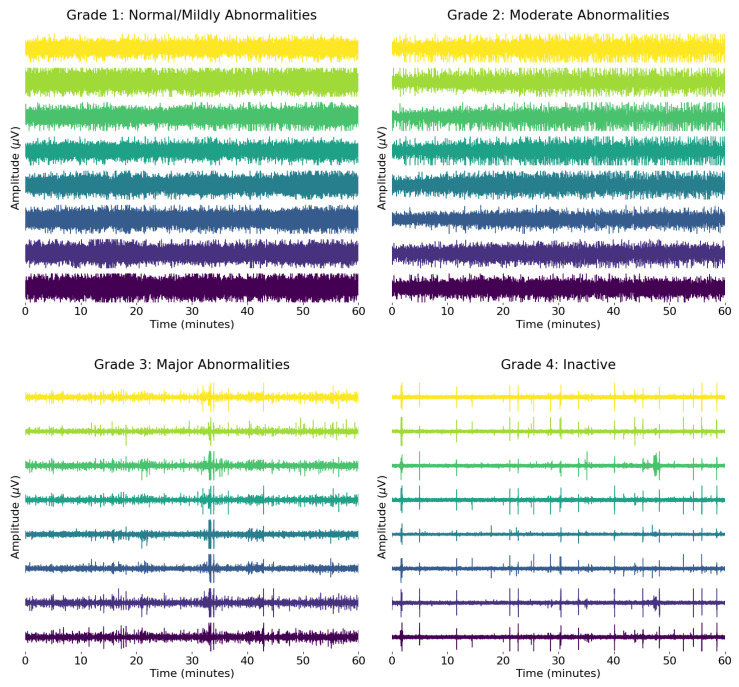
Bipolar montage representation of the EEG signals for each HIE grade. A total of 60 min of the EEG signal is plotted, and the amplitude scale for each signal is in the range of +50 (μV) and −50 (μV).

To initiate this treatment within the first 6 h of the neonate’s life, an efficient and accurate diagnosis is essential. In order to diagnose the patient, a multi-model approach is used in a clinical setting. This includes, but is not limited to, physical examination, neuroimaging analysis, electrophysical studies, and biomarker analysis [12]. Each of these diagnostic methods has its own limitations, particularly within the critical early hours when timely intervention is essential. Physical signs, such as abnormal pupil reactivity and motor responses, are more predictive after 24 h post-injury [12], while MRI and DWI, although effective at identifying subacute lesions, often fail to detect minor brain injuries and are not always feasible for unstable neonates [13,14]. Biomarkers have shown early promise [4], but they are not yet fully reliable as standalone indicators. In contrast, Electroencephalogram (EEG) signals have been found to be beneficial in monitoring the neurological status of neonates [15,16], with the highest sensitivity (0.93) and specificity (0.90) found across all modalities for HIE grading [13]. Figure 1 shows the bipolar montage representation of the eight EEG signal channels for all four HIE grades. This figure aligns with the findings in [13], where it is clear from reading the EEG signal that the continuous brain activity pattern degrades as severity increases from grades 1 to 4 and thus making accurate diagnosis possible based on the EEG signals.

In addition, access to EEG monitoring devices has been found widespread in neonatal care units across the globe [17]. Despite these benefits, studies have shown that complex EEG signals, which are true for EEG signals of patients with HIE, can take anywhere from one seventh up to half the time of the original EEG signal to analyze [18]. This presents a significant challenge for medical professionals, as the extended time required for manual grading may delay critical interventions. This underscores the pressing need for more efficient and accurate grading methods to support timely diagnosis and intervention within the critical therapeutic window.

To address this issue, significant research efforts have been directed towards developing automated grading systems, with a primary focus on analyzing neonatal EEG signals [19,20,21]. Early research in this field started with exploration of the amplitude of the EEG signal and its relationship with discontinuity patterns based on quantitative EEG feature analysis [21,22]. Lacan et al. [22] identified these features as the primary focus for visual grading of EEG signals, both based on human interpretation and automated grading methods. An accuracy of 79.5% was achieved by Guo et al. [21], building upon this work, processing the quantitative EEG features for 64 s windows classified using Support-Vector Machines (SVM). Ahmed et al used a supervector approach for the task [19]. This included the combination of Gaussian Mixture Models (GMM) with SVMs, where an overall accuracy of 87% was achieved. Raurale et al achieved a leave-one-out (LOO) test accuracy of 88.9%, when using time-frequency distribution (TFD) to extract the features of a 5 min EEG segment, prior to processing these features with a convolutional neural network (CNN). This accuracy, however, fell to 69.5% on a large unseen private dataset consisting of 338 h of patient data. Utilizing fully convolutional layers for both the feature extraction and classification process based on a 60 s window of the EEG signal, Yu et al. [23] achieved a 23.65% increase in the accuracy on this large unseen dataset with a test accuracy of 86.09%.

This research to date has shown the transition from traditional feature extraction methods to end-to-end neural network (NN)-based systems and has set a new benchmark, with the potential for further improvements in the future. The analysis by Raurale et al. in [20] has reported the use of Convolutional Neural Networks (CNN) for HIE grading when representing the EEG signal as a 2D spectrogram image. The image representation of the EEG signal has been widely used for various EEG-related tasks, including seizure detection [24,25] and classification of other neurological patterns [26], motivating its adaptation here for acute neonatal brain injury. In the recent Kaggle competition, Harmful Brain Activity Classification [27], all winning approaches utilized image recognition backbones to help with the classification of image-based representation of EEG. The high performance achieved for these tasks highlights the benefits of spectrogram representation of the EEG signal, which is rich in information, describing the EEG signal in both the time and frequency domains. Spectrogram-based CNNs have shown to be on par with and surpass the performance of 1-dimensional networks in arrhythmia detection based on ECG signals [28,29,30]. This is an important finding from the literature as it highlights the potential of 2D CNNs in identifying rhythmic behavior from spectrogram representations of pathological signals. This is particularly relevant for HIE grading, as one of the primary measures seen to show accurate HIE grading is the continuity pattern of the signal [20,31].

While the ability to identify rhythmic behavior of the signal is important, the size of the input window has been a significant contributor to the accuracy of the grading system [20,23]. While some studies have shown 20 min windows to be sufficient [32], the standard window of interest in automated grading systems is 1 h [19,31,33]. This provides a balance between capturing sufficient data for clinical assessment and allowing for timely clinical decisions. Current automated grading methods in the literature look at either averaging the features over the hour epoch during the preprocessing phase [31] or using postprocessing aggregation as seen in [23]. Advanced signal processing techniques are required to increase the window size of the EEG input and maintain the accuracy performance without overwhelming system resources.

One promising approach is sonification, which offers a method of compressing the EEG signal without losing critical information. By transforming the signal into the audio domain, sonification enables the input of longer EEG sequences into the CNN, ensuring that the rich temporal structure of the signal is preserved while reducing the computational load. This allows the full EEG signal to be processed efficiently, offering the potential to leverage larger input windows without sacrificing accuracy or speed. The use of sonification has already demonstrated significant benefits in seizure detection systems, where audio domain representation increases accuracy and requires minimal training of the medical professional to interpret complex signals [34,35,36]. While its potential in seizure detection has been studied, its application to HIE grading remains unexplored.

Chalak et al. [37] suggested caution against offering therapeutic hypothermia for neonates with Grade 1. Previous work on HIE grading has highlighted that the grades which are most relevant for therapeutic intervention, Grade 1 and Grade 2, are also the most challenging in differentiating. In [23], the AUCs of Grades 1 and 2 were 0.87 and 0.73, whereas Grades 3 and 4 reached 0.9. an 0.94. A similar pattern was also demonstrated in [19,20] with the confusion matrix indicating the most errors between Grade 1 and Grade 2. Nathan et al. [38] has demonstrated that Grade 1 and Grade 2 are also the most challenging from the human annotation perspective by analyzing the human interobserver agreement when grading long-term EEG.

This paper proposes a novel method for HIE grading by combining the potential of sonification as a signal representation technique with 2D CNNs for spectrogram signal analysis. The work has a particular focus on the improvement of grading accuracy between Grades 1 and 2, which are the most relevant for therapeutic intervention. The main technical contributions of the study towards that aim are:

- A novel method of long-term EEG representation by the usage of frequency and amplitude modulation data transformation, applied only for short-term EEG seizure detection prior to this research.
- A novel ML modeling pipeline that accurately models long-term EEG compressed spectrograms to leverage computer vision backbones, formulating the problem as a regression task (as opposed to classification) to leverage the monotonic relationship between grades.
- A novel postprocessing technique to convert regression values to grades based on optimized rounder thresholds.

Unlike previous work, the whole system is built to model long-term EEG without requiring any temporal postprocessing or smoothing. The code and the models are made publicly available to ensure comparison with the future innovations in the area, (https://github.com/leahtwomey/Long_term_EEG_Grading_Using_DSP_ML, accessed on 8 May 2025).

## 2. Methods

The following section of the paper details all aspects of the proposed method and the techniques used to evaluate its performance. In order to achieve a well-developed autonomous HIE grading method, a high-quality dataset is required.

### 2.1. Dataset

The publicly available dataset by O’Toole et al. [39,40] is used in this study, which contains hour-long segments of EEG signals from 53 neonates. A total of 169 1-h EEG segments were collected. The patients in the dataset have a median gestational age of 40 weeks. Each recording was collected within the first 48 h after birth. The NicoletOne ICU and the Neurofax EEG-1200 EEG monitors were used with a sampling rate of 200 Hz and 256 Hz, respectively. The EEG was recorded with the 10:20 system used for electrode placement [39], with 8-channel bipolar montage used for analysis.

The dataset was graded according to Murray et al. [41], with Grades 0 and 1 by definition [41], then grouped into a single new Grade 1 classification for this public dataset. The four severity grades within the dataset are Grade 1, 2, 3, and 4 represented by 61.54% (104 h), 18.34% (31 h), 13.02% (22 h), and 7.1% (12 h), respectively [39]. Independent grading was conducted by two clinical physiologists, and any discrepancies were resolved by consensus.

The dataset defines the grades as follows, where Grade 1 represents normal/mild abnormalities, Grade 2 indicates moderate abnormalities, Grade 3 is severe abnormalities, and Grade 4 represents isoelectric signals.

### 2.2. Proposed Workflow

**Figure 2:**
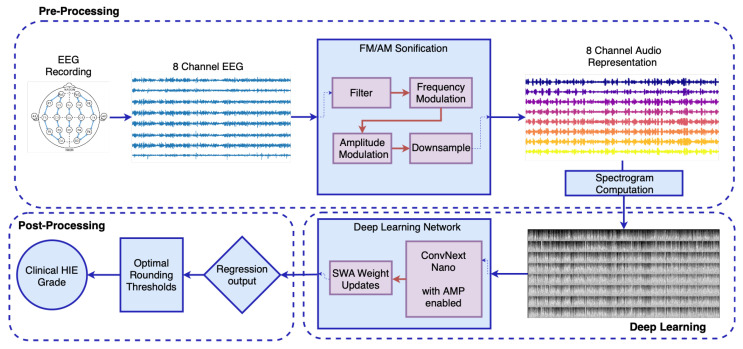
Proposed workflow for a novel method to autonomous HIE grading.

The proposed method, illustrated in Figure 2, integrates advanced signal processing techniques with deep learning to improve HIE grading accuracy. The technical configurations across the preprocessing, neural network, and postprocessing stages are detailed in this section.

#### 2.2.1. Signal Preprocessing

Conversion of the EEG signal to the audio domain representation is used to enhance the interpretability of the signal, leveraging an adaptation to the frequency and amplitude modulation (FM/AM) sonification algorithm first proposed by Gomez et al. [34]. This technique, previously applied to neonatal seizure detection, is utilized here to highlight the rhythmic patterns in background EEG signals related to HIE severity. The sonification algorithm is detailed below, providing an overview of the processing steps, with an additional downsampling step added to the original sonification algorithm.

1. Preprocessing: The EEG signal is filtered between 0.5 to 7.5 Hz after the implementation of a notch filter and downsampled to reduce computational load [34]. This was carried out to preserve rhythmic activity predominantly found in the delta and theta bands of the neonatal EEG [42,43], while accommodating the constraints introduced by downsampling. The upper cutoff at 7.5 Hz was chosen to allow for an effective anti-aliasing filter with a reasonable transition band before the Nyquist limit, avoiding the need for an excessively sharp filter design. This trade-off ensures minimal loss of relevant signal content while maintaining computational efficiency and filter stability. Dynamic range compression is applied to the amplitude of the signal to prevent distortion during the frequency modulation (FM) stage. An envelope is applied to capture the signal energy, with the envelope compressed for any values exceeding a pre-defined threshold of −20 dB.
2. Frequency Modulation: A carrier sinusoid centered at 500 Hz is modulated based on the processed EEG signal, with an exponential transform then applied to convert the EEG frequencies to semitones, following the musical definition of an octave.
3. Amplitude Modulation: The FM signal is then modulated with the envelope of the EEG signal, emphasizing long-term rhythmic patterns in the EEG, a critical feature for defining the EEG grade.
4. Downsampling: The audio signal is downsampled, based on Fourier Transform interpolation and satisfying the Shannon-Nyquist Sampling Theorem [44] to a frequency of 512 Hz.

This sonification method leverages time compression and downsampling to retain the core rhythmic and amplitude features, allowing for an extended temporal window to be represented concisely. This processing choice ensures that only the most relevant signal information is preserved while enabling more manageable, focused analysis in both time and frequency domains. It was seen in [34] that increased speeds showed improved accuracy at identifying seizures in the sound domain, and thus this observation was used in our methodology, where a compression rate of 20 is applied. Each one-hour epoch of EEG data is compressed and represented as approximately 3 min in the audio domain.

**Figure 3:**
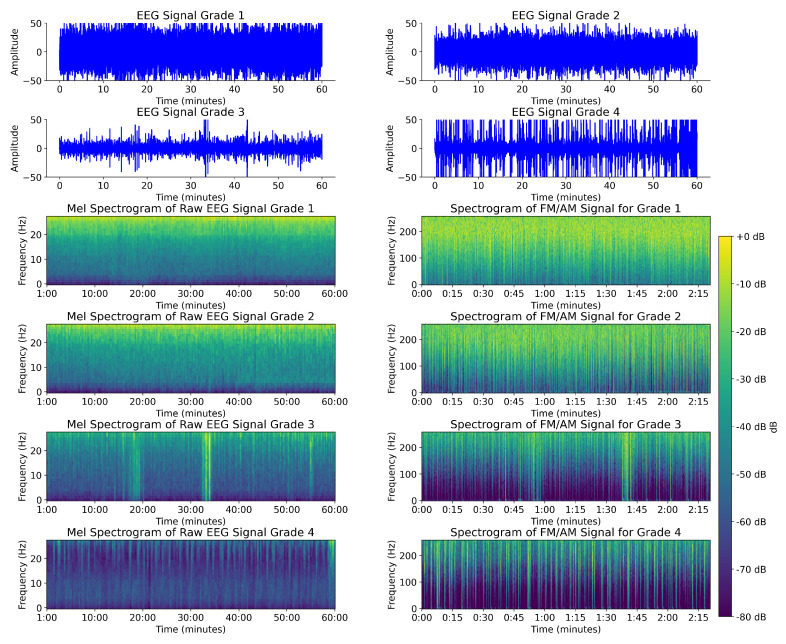
Raw EEG signal plot for different EEG grades (top four plots), Mel spectrogram of EEG signal (**right**) and Spectrograms of the FM/AM transformed EEG signals for different EEG grades (**left**).

Following the sonification, the final step in the preprocessing stage is the spectrogram computation of the audio signal. The suitable spectrogram parameters were an FFT window length/shift of 128/64. A Hanning window was applied to each segment to minimize the signal discontinuities at the beginning and end of each windowed segment. This results in a time resolution of 125 milliseconds and a frequency resolution of 4 Hz. To summarize the preprocessing transformations, the EEG signal ((EEG channels × fs × duration) e.g., 8 × 256 × 3600) is converted first with FM/AM to 8 × 512 × 180. After the spectrogram, it is transformed into 8 × 65 × 1126. The resultant spectrogram representation of the EEG for a single-channel sample for each EEG grade is shown in Figure 3. Apart from the distinct energy level, the image also conveys the richness of the frequency content. The differences among the grades are visually identifiable in the figure, suggesting they offer differentiability that can enhance the classification task based on visual inspection, which is a key objective of this preprocessing stage.

Spectrograms of the raw EEG signal are needed for comparison with the FM/AM transformed signal in order to visually inspect the improvements in differentiating the HIE grades based on the signal representation. There are many methods to represent time-frequency as an image, including Mel spectrogram [45], continuous wavelet transform (CWT) [46], Hilber-Huang Transform (HHT) [47], S-transform [48], and least-squares wavelet analysis (LSWA) [49]. Many methods of spectrogram generation have been tried in recent literature, including the latest Kaggle competition [27]. However, a simple Mel spectrogram was a common feature of most of the winning methods in this competition. One reason for this is that the Mel scale reflects human auditory perception, emphasizing lower frequencies, and it is these lower frequencies that are of high importance for EEG signal analysis.

Comparing the spectrogram of the FM/AM transformed signal with the Mel spectrogram of the original EEG signal in Figure 3, the visually differentiating features of the signal are not present. While grade 4 is easy to identify in Figure 3, the remaining three grades do not contain the same features as the spectrogram of the FM/AM transformed signal in Figure 3.

**Figure 4:**
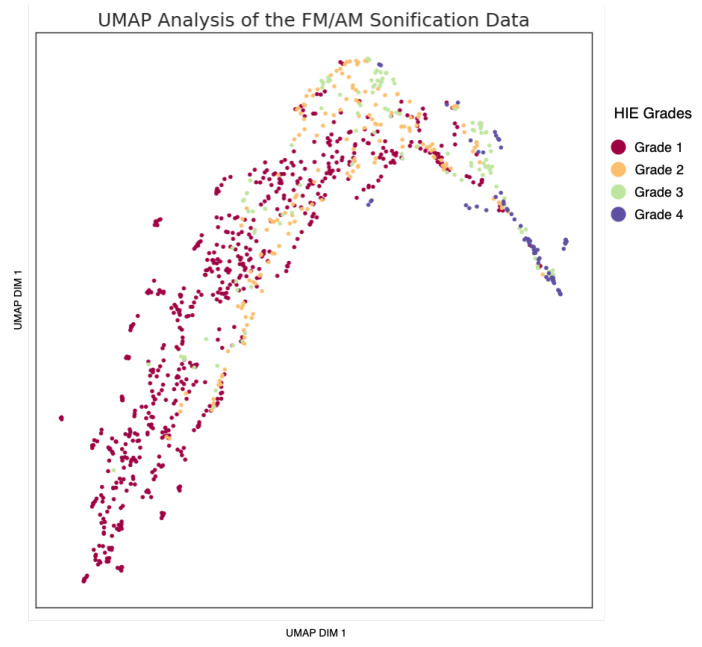
UMAP plot of the FM/AM transformed EEG signal on an individual channel basis across the four EEG grades.

Uniform Manifold Analysis Projection (UMAP) [50], projection of the images into a lower-dimensional representation is presented in Figure 4. The UMAP plot highlights meaningful separability that aligns with our classification objectives.

**Figure 5:**
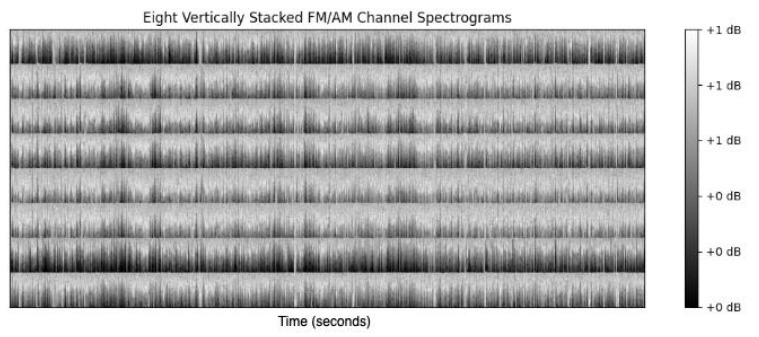
Vertically stacked spectrograms of eight EEG bipolar montage channels, sonified using the FM/AM modulation method.

The final input image to the CNN vertically stacks the spectrograms of each channel, as seen in Figure 5. By vertically stacking spectrograms for each channel, we achieve an integrative visual representation that underscores cross-channel rhythmic patterns critical to accurate HIE grading, resulting in improved model performance by focusing on the core attributes of interest across channels.

#### 2.2.2. Deep Learning Model

Stacking eight FM/AM transformed EEG spectrograms forms a 2D representation that is well suited for computer vision image recognition backbones. The ConvNext architecture [51] was selected to operate on these resultant images to grade EEG. The pretrained ConvNextV1 Nano architecture [52] was trained on spectrogram images of FM/AM transformed EEG signals. The Nano version was chosen as it showed a suitable trade-off between model complexity and the dataset size.

A regression head was attached to the model, following a global average pooling function. In contrast, the classification head that can be used to model 4 classes would consider the classes to be independent and would not be able to leverage and exploit the monotonic relationship between the grades.

Cosine annealing with warm restarts was implemented as the learning rate scheduler [53]. This helps to improve the depth of learning and allows the surpassing of local minima without random reinitialization of the weights. To enhance the generalization of the model, Stochastic Weight Averaging (SWA) was implemented in addition to the Adam optimizer, successfully stabilizing the weight fluctuations [54]. Automatic Mixed Precision (AMP) was utilized to optimize the memory usage and computational speed of the model [55]. This reduced training time by 41.3% and memory usage by approximately 11 GB without compromising accuracy. AMP’s dynamic approach ensured stability and efficiency during training.

#### 2.2.3. Postprocessing

An optimized rounder class was implemented as a postprocessing step to convert the continuous regression value of the model to a discrete clinical grade. The idea was borrowed from the PetFinder Kaggle competition [56], where it was first utilized to convert the classification problem with the monotonic class relationship into regression. Optimal rounding thresholds which best separate the continuous predictions into their respective classes are computed based on Nelder-Mead Optimization [57]. Nelder-Mead is a popular method for optimization and is a minimization algorithm, which seeks to make the difference between the predicted value and the target value as small as possible [57]. The optimized rounder is designed to identify a set of C−1 thresholds such that the continuous predictions are separated into C discrete classes with a prespecified objective metric. By fine-tuning the rounding thresholds, the alignment between the predicted and true class is enhanced, thus improving the model’s overall performance.

### 2.3. Performance Metrics

The model performance before postprocessing was assessed using the Mean-Squared Error (MSE) and the R-squared ($R^{2}$), and after postprocessing using accuracy, precision, recall, F1-score, and Cohen’s Kappa. MSE quantifies the average squared difference between the true and predicted values, where a lower MSE indicates a better model fit [58]. Hyperparameter tuning during model selection was performed mostly using MSE loss as the main metric. The $R^{2}$ value is indicative of the proportion of variance in the target variable that is explained by the model. A value of 1 represents a perfect fit [59]. Precision measures the proportion of true positives out of all positive predictions [60], while recall assesses the model’s ability to correctly identify all relevant positive instances [61]. The F1-score, the harmonic mean of precision and recall, balances these two metrics [62]. Cohen’s Kappa score was used as a robust metric to measure agreement between the predicted and actual values, accounting for chance agreement. A Kappa score of 1 indicates perfect agreement, while 0 indicates agreement no better than chance [63].

A one-vs-rest approach is applied to compute the Receiver Operating Characteristic (ROC) curve and the corresponding Area Under the Curve (AUC) for each class. Each class is treated as a positive class, while all other classes are combined into a single negative class. To compute the ROC curve, a scoring metric is defined based on the distance between the predicted value and the target class. The score is calculated as 1−|predicted−class|, reflecting the proximity of the prediction to the target class in order to rank the model’s outputs.

UMAP analysis for the feature map of the proposed method, prior to the global average pooling and linear regression header, is assessed, and a comparison of the UMAP embedding clustering is performed. The Davies-Bouldin Index (DBI) [64], the Silhouette score [54], and the Calinski–Harabasz Index (CHI) [65] are used to assess the clustering at both a local and global level for each UMAP projection. DBI assesses the clustering quality by comparing the average similarity between the pairwise most similar clusters, with a lower DBI value indicating a better clustering solution. Silhouette score is a measure of how similar an object is to its own cluster compared to other clusters and ranges from −1 to +1, with a high value indicating that the object is well matched to its own cluster. CHI measures the spread of datapoints between clusters vs within clusters, with a higher value of CH indicating a better clustering.

### 2.4. Nested Cross-Validation Evaluation Framework

**Figure 6:**
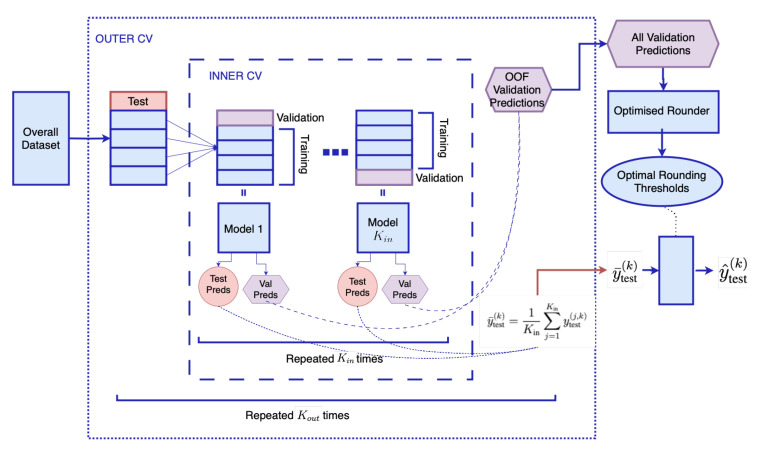
Nested Cross-Validation Evaluation Framework.

To mitigate the risk of overfitting due to the limited dataset size, a robust nested cross-validation (CV) approach was implemented for performance assessment. This step is computationally expensive. However, it allows the attenuation of the effect of random noise on performance metrics and enhances the sensitivity of the experimental setup to the hypothesis being tested. In addition, this framework also ensures hyperparameter tuning is performed only within the inner folds. This leads to a more efficient and structured model selection process. A nested CV has been proven to provide unbiased performance metrics. An overview of the evaluation framework implemented is provided in Figure 6. A 5-fold outer loop and 4-fold inner loop Stratified Group K Fold Cross-Validation are implemented [66]. This ensures a patient-independent model is developed by grouping, which is based on patient IDs. Given the skewed distribution across classes discussed previously, a stratified split was crucial to preserve the distribution across folds while preserving patient integrity. The model was trained on subsets and validated within the inner folds, with the test performance evaluated on the unseen samples from the outer fold. For each outer fold, an ensemble of deep learning models is built, and the out-of-fold (OOF) validation predictions are used to train the optimized rounder for identifying the suitable rounding thresholds to define each clinical grade. The ensemble of models is then tested on the outer fold, and the test predictions for each sample are averaged. The continuous value is converted to a discrete clinical grade by the tuned rounding thresholds. The overall model performance was derived by aggregating accuracies across all outer folds, ensuring an unbiased and comprehensive evaluation of the model’s generalization capability on unseen data. The overall accuracy of the model is calculated as per Equation (1). The overall accuracy is computed by summing the number of correct predictions made on the outer test folds and dividing by the total number of samples. For each fold, a prediction is considered correct if it matches the true label. This approach ensures that the performance estimate is based only on unseen data, giving an unbiased view of how the model would perform in a real-world setting.

(1) $$\mathrm{Accuracy}=\frac{1}{N}\sum_{k=1}^{K_{\mathrm{out}}}\sum_{i\in D_{\mathrm{test}}^{\left(k\right)}}I\left({\hat{y}}_{\mathrm{test},i}^{\left(k\right)}=y_{\mathrm{true},i}^{\left(k\right)}\right)$$

where $K_{out}$ is the number of outer folds, 5 in the case of this study, ${\hat{y}}_{\mathrm{test},i}^{\left(k\right)}$ is the average prediction for the test sample *i* across all inner folds, $y_{\mathrm{true},i}^{\left(k\right)}$ is the ground truth value for test subject *i* and *N* is the number of samples in the overall dataset. $D_{\mathrm{test}}^{\left(k\right)}$ represents the set of test samples in the *k*-th outer fold, and I(·) is the indicator function, which returns 1 if the predicted label matches the true label, and 0 otherwise.

## 3. Results

The proposed method achieved a test accuracy of 89.97%. A precision and recall score of 0.9079 and 0.8994 was achieved, respectively, showing a high predictive capability of the model. The F1-score of 0.8985 showed the model’s overall ability to correctly identify positive cases. The $R^{2}$ values indicated that 85.07% of the variance in the target variable can be explained by the model. A strong agreement beyond random chance is achieved based on Cohen’s Kappa score of 0.82.

**Figure 7:**
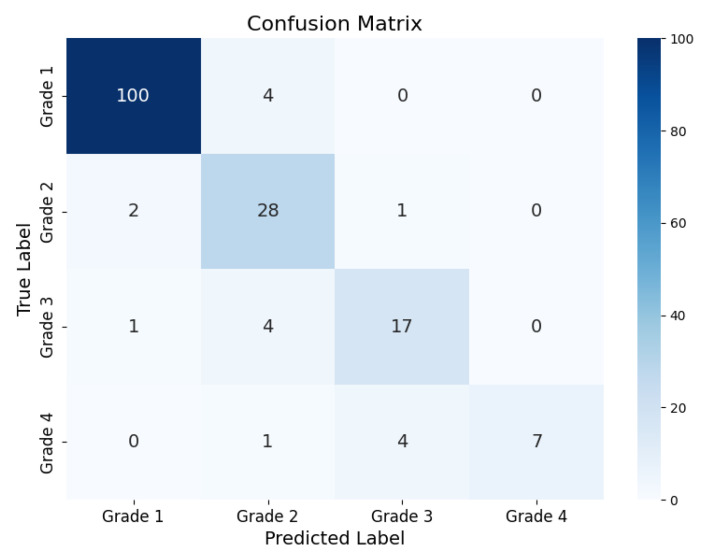
Confusion Matrix Representing Test Results of the Nested Cross-Validation Evaluation Framework.

Figure 7 shows a confusion matrix. The model performs the best at correctly identifying Grade 1 samples. Correctly predicting the remaining three grades was increasingly more challenging. The accuracies across grades were 96.2%, 90.3%, 77.3%, and 58.3%, for Grades 1, 2, 3, and 4, respectively. The distance-based AUC scores are 0.98, 0.96, 0.93, and 0.97 for Grades 1, 2, 3, and 4, respectively.

**Figure 8:**
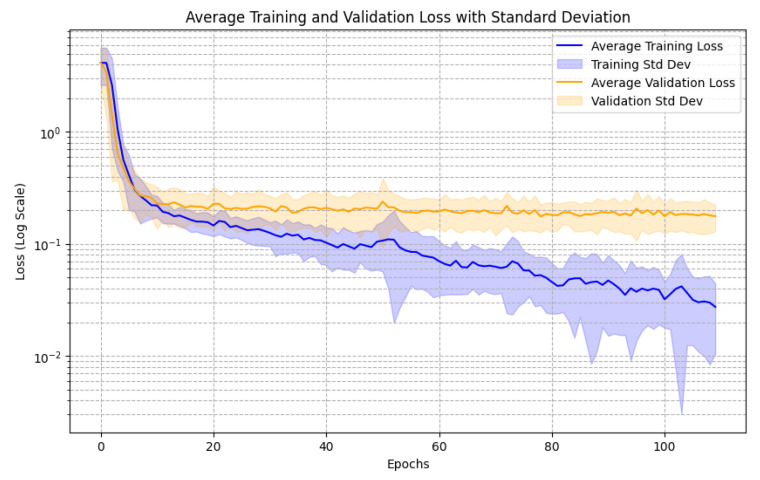
Nested cross-validation learning curves showing training and validation loss. The *x*-axis indicates the number of training epochs, while the *y*-axis represents the loss (logarithmic scale). The solid blue and orange lines show the average training and validation loss across all folds, respectively. Shaded regions represent the standard deviation of loss at each epoch, reflecting variability across folds.

The training and validation MSE loss learning curves are seen in Figure 8. The average learning curve across all folds is plotted, and the standard deviation across folds is seen as the masking shadow on the plot.

**Figure 9:**
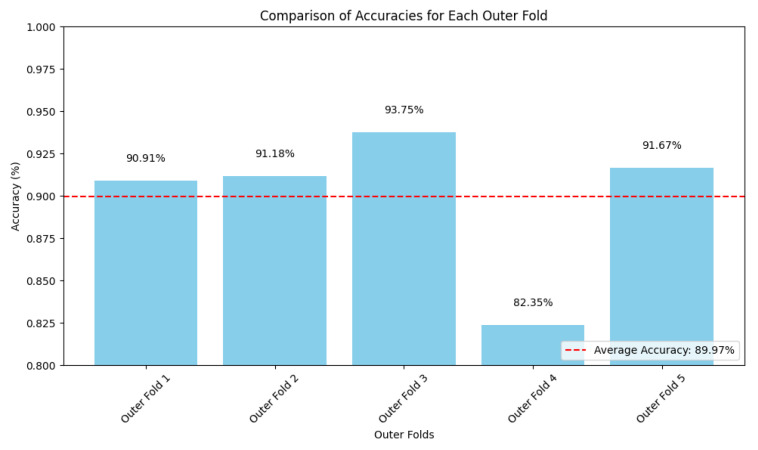
Test Accuracy for Each Outer Fold in Nested Cross-Validation Evaluation Scheme.

**Figure 10:**
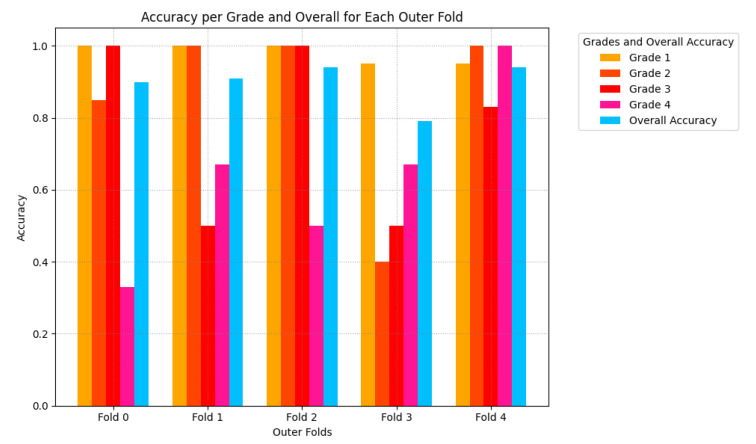
Accuracy Per Grade per Fold for each Outer Fold.

The average accuracy for each fold is depicted in Figure 9 with Figure 10 showing the distribution of accuracy per grade.

The incorrectly predicted samples of outer fold 4 were evaluated. Their predictions for each validation and test runtime in the nested cross-validation evaluation framework are provided in Table 1. Table 1 shows that for the given 6 samples, incorrectly predicted in outer fold 4, the model failed to accurately predict these samples both in the validation and test scenarios. This suggests that these samples may contain outlying features, which resulted in difficulty for the model predicting their true grade.

Table 2 states the performance metrics for the model trained on the FM/AM transformed spectrograms and the EEG signal Mel spectrograms. This comparative test was conducted to evaluate the benefit of using the FM/AM transformation in terms of EEG grading accuracy. A clear improvement across all performance metrics is noted in the model performance when using the FM/AM transformed spectrogram, with the overall accuracy increasing by approximately 10% to a value of 89.97%.

The FM/AM transformed EEG spectrogram resulted in consistently higher performance than the EEG Mel spectrogram model. The EEG signal was processed with the same filtering process for both representations. A statistically significant difference between the two methods is achieved based on the Wilcoxon signed-rank test, where a *p*-value of 0.03 is achieved.

The proposed method converts this problem to a regression task to leverage the monotonic relationship between the grades. Model performance was evaluated in classification mode to assess whether using a regression approach, intended to leverage the relationships between grades, led to the anticipated improvement. Table 3 provides the results of the regression versus classification implementation of the model. For this comparative test, a fixed data split, maintaining patient independence and stratification, is used to ensure comparability. It is clear from Table 3 that the regression model performs better in terms of accuracy performance in training, validation, and test over the classification method.

**Figure 11:**
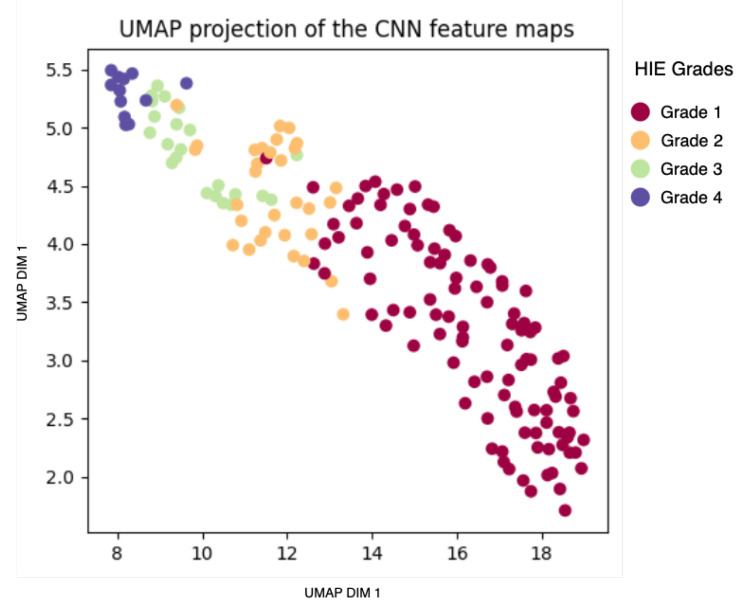
UMAP plot of the feature map across the four EEG grades.

The feature map of the proposed method is extracted prior to the global average pooling layer. UMAP analysis is performed to evaluate the separability performance of the model. Figure 11 plots the UMAP embedding for a single fold. It can be seen that the chosen representation indeed highlights the difference between Grade 1 and Grade 2, whereas the other grades have larger overlaps in their representation.

The clustering behavior of the UMAP embedding is assessed for the EEG signal, the FM/AM transformed signal, and the feature map of the proposed method. The results of this comparative analysis are seen in Table 4, where the DBI, CHI, and Silhouette score are used as the metrics of evaluation. The goal of this experiment was to evaluate the clustering performance of the grades at three different phases of the proposed pipeline. Firstly, the raw EEG signal prior to any preprocessing; secondly, after the FM/AM data transformation; and finally, after the signal has passed through the CNN layers. The results in Table 4 show significant improvement in clustering for the feature map output of the CNN, with these results further evaluated in the discussion in Section 4.5.

## 4. Discussion

### 4.1. New State of the Art

The proposed method achieves a new state-of-the-art accuracy of 89.97%. The confusion matrix in Figure 7 shows the model performs very well at identifying Grades 1 and 2. This is further supported by the grade-wise accuracy seen in Figure 10 along with the high AUC scores for Grades 1 and 2. Identifying these grades is very important for initiating therapeutic treatment, as caution is advised for whole-body hypothermia for neonates with mild HIE [37]. The proposed method results in an improved performance focused particularly on Grades 1 and 2. This finding highlights the potential for the proposed method to complement existing approaches in the literature, where the preprocessing phase of the proposed method emphasizes the differences between Grades 1 and 2. For instance, grade 4 could be detected effectively using simpler methods such as energy thresholding, while Grades 2 and 3 could benefit from the nuanced feature extraction capability of the proposed deep learning approach.

This method uses a large input window of 1-h EEG signal, providing the model with a strong understanding of the global features of the signal, which define the EEG grade. Other methods in the literature look at segmenting the EEG signal into smaller windows [20] or use postprocessing techniques to aggregate the predictions over time [23].

Representation of the FM/AM transformed data in the image domain required identifying optimal FFT window/shift length parameters. An experimental grid search was performed, with the best-performing image being highly rectangular. Given the receptive field of the ConvNextV1 Nano architecture is 1328, it allowed for high performance by capturing global patterns across the spectrogram and learn long-range dependencies within the data despite this rectangular shape. Vertical stacking of the spectrograms for the 8 bipolar channels resulted in the best performance, with random channel shuffling added in the data loader to avoid any potential overfitting during model training.

Figure 8 shows that the validation loss plateaus but does not rise. This indicates that the validation data could have been safely added to train since a model checkpoint anywhere between 60 and 100 epochs would be equally well performing. In our study, however, OOF predictions on validation data served an additional purpose of training the optimized rounder thresholds.

Asymmetry is present in the normalized confusion matrix of Figure 7. The sum of the elements above the diagonal is smaller than the analogous elements below the diagonal. This indicates that if an error is present, the error would typically tend to underestimate the EEG grade rather than overestimate it. It is important to also note that despite the challenges at some grades, only two samples were misclassified to a severity level more than one grade away from their actual label. A test accuracy of 98.82% is achieved based on this tolerance for minor misclassification.

It is well known that EEG signals are inherently susceptible to various artifacts, such as eye blinks, muscle movements, and baseline drift. These artifacts can severely degrade signal quality and, as a result, could have an impact on the grading algorithm. Recent research has put a focus on robust artifact and noise removal, which are crucial for reliable EEG analysis. The baseline correction methods seen in [67] and hybrid artifact removal techniques in [68] have shown promise in improving signal quality prior to classification. While this study has strong accuracy performance, future work will look at enhancing the artifact removal in the signal preprocessing phase to assess its impact on the model robustness.

### 4.2. Comparison Between FM/AM Transformed EEG Spectrogram and Mel Spectrogram EEG

The comparative results in Table 2 demonstrate the advantage of using the FM/AM transformation spectrogram in comparison to the Mel spectrogram of the EEG signal. The improved performance can be attributed to the enhanced differentiability of EEG grades due to the FM/AM transformation. This transformation highlights both the amplitude variation and rhythmic patterns in the EEG signal, two key features clinicians typically assess when grading the EEG signal based on visual analysis. In addition, the proposed method uses a window size of 1-h EEG segments. The FM/AM transformation aids in representing this large duration of data while maintaining the spatial features and frequency resolution of the spectrogram image. It is clear from Figure 3 that the sharpness and clarity of the signal’s frequency component are enhanced for the 1-h EEG segment when represented by the FM/AM transformation. It is important to note that other spectrogram representations of the EEG signal were tested, and the Mel spectrogram returned the highest performance after the FM/AM transformation.

### 4.3. Regression vs. Classification Performance

When designing the deep learning model configuration, a regression model was chosen due to its improved generalized performance. Table 3 showed improved test and validation accuracy for the regression model in comparison to the classification configuration. This is due to the model’s ability to learn the relationships between the features of each grade when addressed as a regression task. In comparison, the classification model considers each grade to be independent, which and so not reflective of the clinical diagnosis.

There are a number of ways in which the implementation of this problem as a regression task can be handled. This study used an optimized rounding function to return a clinical grading. This learns the relationships between the models’ regression prediction and the true clinical grade for the validation data. Optimal thresholds are then identified and applied to the test predictions.

### 4.4. Analysis of Errors

Figure 9 shows outer fold 4 to be an outlier, with an accuracy of approximately 82%. This is a relative decrease of over 8% from the average test accuracy. All other folds show higher accuracy, with little fluctuation across folds. This decrease in outer fold 4 raised concern, and so a significant analysis into the model performance was conducted. With no significant reduction in training or validation accuracy for outer fold 4, it was hypothesized that outliers in the test set were the cause.

A similar performance drop for the EEG Mel spectrograms and the FM/AM transformed spectrograms was noted. This consistency indicated that the sonification method did not worsen the representation of HIE features for these outlier samples. This was an important finding as it confirmed that the FM/AM transformation was effective for the entire dataset.

Grade-wise accuracy analysis revealed a notable drop for Grades 2 and 3 in fold 4. This is clearly seen in Figure 10. To further investigate these outliers, the incorrectly predicted samples in outer fold 4’s test set were evaluated in Table 1. The model struggled to correctly predict these samples both during validation and testing for different folds. This indicates that these samples likely contained outlying features. While the dataset states there are two annotators, only the consensus result is provided. Access to this individual annotator information would be useful to clarify the outliers in the dataset and assess whether these were subject to disagreement between the annotators.

In order to evaluate the effect these outlier samples have on the model performance, the test accuracy excluding fold 4 is calculated. A test accuracy of 92.59% is achieved, highlighting the limiting effect on the performance of these outliers had.

### 4.5. Analysis of Model-Based Representation

UMAP analysis is used to examine the separability performance of the proposed method. The goal was to evaluate the benefit of the proposed method for HIE grading. An improvement in separability is obvious when comparing the UMAP embeddings in Figure 4 and Figure 11. This highlights the improved separation based on the CNN. This suggests the CNN has effectively extracted meaningful features that represent the variations among the EEG grades. In addition, it supports the need for the complex CNN architecture to perform this task, as the HIE features are highly non-linear, making it a complex task.

Looking at the quantitative metrics, in Table 4, in addition to the visual inspection of the plots, it is clear that the feature map of the proposed method showed overall best performance of best performance of separability and clustering. With a DBI of 0.8, it significantly outperforms both the raw EEG (DBI = 14.6) and sonified EEG (DBI = 2.9). This indicates much better compactness and separation of clusters. Similarly, the feature map’s Silhouette Score of 0.3 marks a substantial improvement over the raw EEG (−0.26) and sonified EEG (0.06). This further confirms the CNN model’s ability to learn discriminative features. However, a lower CHI is measured for the feature map (166) than that of the sonified EEG (224). This can be attributed to the denser representation of the sonified data, due to the sonified data being evaluated on a single-channel basis. Overall, the feature map representation demonstrates superior cluster quality compared to both raw and sonified EEG data, highlighting the effectiveness of the proposed method.

### 4.6. Methodology Refinement and Experimental Justification

The input data configuration and model parameters were carefully selected after experimental testing and evaluation. A single-channel model was initially developed with the CNN output concatenated for the eight EEG channels. This representation meant that information regarding the cross-channel relationships was not obtained by the model. In addition, it was impractical as it required eight inference run times for a single evaluation. Concatenating the channel spectrograms was then evaluated, both in the image domain as horizontal and vertical concatenation, and in the channel domain. Horizontal concatenation resulted in a largely rectangular input image, and concatenation in the channel domain showed no significant improvement in performance over the single-channel model. This experimental testing showed the benefit of stacking the channel spectrograms in the vertical axis of the image domain as a data input representation. In addition to testing different concatenation representations of the spectrogram, different spectrogram parameters were evaluated with internal cross-validation used to identify the optimal parameters. These included looking at the window/shift relationship of 128/64, 64/32, 256/128, 512/256, and 1024/256. With best results from our internal experiments were achieved with 128/64.

ConvNext Nano is a well-known CNN model, which has shown strong performance in image recognition tasks to date and has shown superior results for the proposed EEG grading task. However, it was not the only backbone architecture tested for this task. Evaluation of different models within the ConvNext architecture family was tested, which included assessing the impact on model size in comparison to grading accuracy. This experimental analysis showed that larger models resulted in overfitting to the training data due to the limited size of the public dataset. In addition, models such as ResNet and EfficientNet architecture types were tested, but superior performance was seen in internal cross-validation testing for ConvNext Nano, thus supporting its selection for this task.

### 4.7. Computational Complexity

The computational cost of the experiment is high only in the training phase. This is due to the nested cross-validation framework, which is detailed in Section 2.4 of this paper. The benefits of this evaluation framework outweigh the computational cost as it results in a highly robust experimental setup. The testing then benefits from the ensemble models with a limited computational cost of the model at this stage.

The preprocessing pipeline, converting the 8 EEG bipolar montage channels to the audio domain signals using FM/AM transformation and then conversion to the spectrogram image, took 0.3311 s per EEG channel. This is a total of 2.648 s for all eight bipolar montage channels. The inference time of the CNN for the one hour of EEG represented as a spectrogram image is 0.29 ms on NVIDIA Quadro GV100 GPU. The postprocessing optimized rounder function takes a total of 0.898 ms to compute. This is a total computational cost for the end-to-end pipeline of 1.09 s.

## 5. Conclusions

This paper presents a novel automated end-to-end EEG grading system for neonates, leveraging signal processing and machine-learning techniques throughout all the phases of the modeling pipeline. By transforming the EEG signal to the audio domain with FM/AM followed by spectrogram computations, the differentiability of the signal between the four EEG grades is highlighted, with the main benefit being the increased separation of Grade 1 and Grade 2, which are the most relevant for the clinical therapeutic decision making. The established image processing backbone is then leveraged to learn the features of the long-term EEG compressed spectrogram and to regress a continuous clinical grade. The optimized rounder is implemented to convert the continuous value to the final grade. An overall test accuracy of 89.97% is achieved, surpassing previous state-of-the-art performance. These findings highlight the potential of using image-based modeling of long-term transformed EEG representation to enhance clinical diagnosis of HIE.

## Figures and Tables

**Table 1:** Comparison of Validation and Test Predictions.

True Value	Validation Predictions (Avg)	Test Predictions (Avg)
4	3.5354, 3.2588, 3.7585, 2.9211 (3.3234)	3.35
2	1.015, 0.982, 1.09, 1.078 (1.0418)	1.079
1	2.0059, 1.0475, 1.7400, 2.22 (1.755)	1.63
1	1.143, 2.4976, 2.387, 2.56 (2.14)	2.504
3	1.0998, 1.625, 1.7237, 1.90576 (1.5887)	1.733
3	2.589, 1.7465, 1.8679, 1.822 (2.0066)	1.746

**Table 2:** Comparison of Model Performance Metrics.

Metric	FM/AM Transformed EEG Spectrogram	EEG Mel Spectrogram
Test Accuracy	89.97%	81.66%
Precision	0.9079	0.7858
Recall	0.8994	0.7576
F1-Score	0.8985	0.7547
R-Squared	0.8507	0.7574
Cohen’s Kappa Score	0.8219	0.5622

**Table 3:** Regression vs. Classification Model Performance.

Metric	Regression Model	Classification Model
Train Accuracy (%)	96.30	94.55
Validation Accuracy (%)	92.31	88.46
Test Accuracy (%)	93.94	90.91

**Table 4:** Clustering Metrics Results.

Metric	Raw EEG	Sonified EEG	Feature Map
Davies-Bouldin Index	14.637	2.985	0.8
Calinski–Harabasz Index	0.8584	224.308	166.8
Silhouette Score	−0.2602	0.06	0.3457

## Data Availability

The dataset used in this manuscript is publicly available at https://zenodo.org/records/7477575, accessed on 8 May 2025. All inference scripts and model weights detailed in this manuscript are available at (https://github.com/leahtwomey/Long_term_EEG_Grading_DSP_ML.git), accessed on 8 May 2025.

## Abbreviations

The following abbreviations are used in this manuscript:

HIE	Hypoxic–Ischemic Encephalopathy
ML	Machine Learning
DSP	Digital Signal Processing
CNN	Convolutional Neural Network
EEG	Electroencephalogram
FM\AM	Frequency and Amplitude Modulation
CV	Cross-Validation
OOF	Out-Of-Fold
MSE	Mean-Squared Error
